# Development of a robust blood‐based multi‐pathway biomarker assay for early screening and classification of Alzheimer’s disease

**DOI:** 10.1002/alz.086053

**Published:** 2025-01-09

**Authors:** Li Ouyang, Yuanbing JIANG, Hyebin Uhm, Fanny C. F. Ip, Ronnie M.N. Lo, Elaine Y.L. Cheng, Xiaoyun Cao, Clara M.C. Tan, Brian C.H. Law, Paula Ortiz‐Romero, Albert Puig‐Pijoan, Aida Fernández‐Lebrero, José Contador, Kin Y Mok, John A. Hardy, Timothy CY Kwok, Vincent Chung Tong Mok, Marc Suárez‐Calvet, Henrik Zetterberg, Amy K.Y. Fu, Nancy Y. Ip

**Affiliations:** ^1^ Hong Kong Center for Neurodegenerative Diseases, Hong Kong Science Park, Hong Kong China; ^2^ Division of Life Science, State Key Laboratory of Molecular Neuroscience, Molecular Neuroscience Center, The Hong Kong University of Science and Technology, Clear Water Bay, Kowloon, Hong Kong China; ^3^ Guangdong Provincial Key Laboratory of Brain Science, Disease and Drug Development, HKUST Shenzhen Research Institute, Shenzhen–Hong Kong Institute of Brain Science, Shenzhen, Guangdong, 518057 China; ^4^ Hospital del Mar Research Institute (IMIM), Barcelona Spain; ^5^ Barcelonaβeta Brain Research Center (BBRC), Pasqual Maragall Foundation, Barcelona Spain; ^6^ Cognitive Decline Unit, Department of Neurology, Hospital Del Mar, Barcelona Spain; ^7^ IMIM (Hospital del Mar Medical Research Institute), Barcelona Spain; ^8^ Medicine Department, Universitat Autònoma de Barcelona, Barcelona Spain; ^9^ ERA‐Net on Cardiovascular Diseases (ERA‐CVD) consortium, Barcelona Spain; ^10^ Department of Medicine and Life Sciences, Universitat Pompeu Fabra, Barcelona Spain; ^11^ Hospital del Mar Research Institute, Barcelona, Barcelona Spain; ^12^ Department of Neurodegenerative Disease, UCL Queen Square Institute of Neurology, London UK; ^13^ UK Dementia Research Institute, University College London, London UK; ^14^ Therese Pei Fong Chow Research Centre for Prevention of Dementia, Division of Geriatrics, Department of Medicine and Therapeutics, The Chinese University of Hong Kong, Shatin, Hong Kong China; ^15^ Lau Tat‐chuen Research Centre of Brain Degenerative Diseases in Chinese, Gerald Choa Neuroscience Institute, Therese Pei Fong Chow Research Centre for Prevention of Dementia, The Chinese University of Hong Kong, Prince of Wales Hospital, Hong Kong SAR Hong Kong; ^16^ Barcelonaβeta Brain Research Center (BBRC), Barcelona Spain; ^17^ Centro de Investigación Biomédica en Red de Fragilidad y Envejecimiento Saludable (CIBERFES), Instituto de Salud Carlos III, Madrid Spain; ^18^ Hong Kong Center for Neurodegenerative Diseases, Hong Kong China; ^19^ Wisconsin Alzheimer's Disease Research Center, School of Medicine and Public Health, University of Wisconsin‐Madison, Madison, WI USA; ^20^ Department of Psychiatry and Neurochemistry, Institute of Neuroscience and Physiology, the Sahlgrenska Academy at the University of Gothenburg, Mölndal Sweden; ^21^ Department of Psychiatry and Neurochemistry, Institute of Neuroscience and Physiology, The Sahlgrenska Academy at the University of Gothenburg, Mölndal Sweden; ^22^ UCL Institute of Neurology Queen Square London UK, London UK

## Abstract

**Background:**

Alzheimer's disease (AD) is a devastating neurodegenerative disease with delayed diagnosis until the manifestation of symptoms. Although the emergence of blood‐based biomarkers offers hope for easy detection of AD, existing AD‐associated blood biomarkers, known as the “blood ATN biomarkers”, mainly capture the pathological hallmarks of AD, overlooking other AD‐associated biological processes such as inflammation and vascular dysfunctions. Therefore, developing a blood‐based biomarker assay that captures dysregulation beyond the ATN biomarkers may help advance early detection and staging of AD, enabling a comprehensive examination of the disease status

**Method:**

We leveraged ultrasensitive proteomic technology to develop a blood‐based, multiplex biomarker assay for AD. This assay simultaneously measures the levels of 21 blood proteins associated with different biological pathways, including neurodegeneration, inflammation, innate immunity, vascular functions, and metabolic activities. Moreover, we developed an AD risk scoring system by integrating the level changes of these 21 proteins in a machine learning‐based model. The performance of this 21‐protein biomarker assay in AD classification and indication of AD‐related endophenotypes was evaluated in three independent cohorts

**Result:**

We showed that this 21‐protein biomarker assay accurately classifies AD (AUC = 0.9407–0.9867) and mild cognitive impairment (MCI) (AUC = 0.8434–0.8945). It also indicates amyloid pathology in Chinese and European populations. Moreover, this assay simultaneously evaluates changes in five biological processes, providing comprehensive assessment of disease status and revealing heterogeneity of AD among individuals. Notably, dysregulations of biological processes upon AD progression are different between Chinese and European populations, particularly in biological pathways related to inflammation and vascular functions.

**Conclusion:**

Our findings demonstrate the utility of a blood‐based multi‐pathway biomarker assay for early screening and staging of AD in clinical settings and provide insights for patient stratification and the development of precision medicine.

